# Supplementary material for: Intersecting distributed networks support convergent linguistic functioning across different languages in bilinguals
Source: Commun Biol. 2023 Jan 25;6:99. doi: 10.1038/s42003-023-04446-5 (PMC9876897; doi:10.1038/s42003-023-04446-5)
Supplement: Supplementary file 2 — Supplementary Information [file 42003_2023_4446_MOESM2_ESM.docx]

**Supplementary Information**

**Intersecting distributed networks support convergent linguistic functioning across different languages in bilinguals**

Shujie Geng^1, 2^, Wanwan Guo^1, 2^, Edmund T. Rolls^1,3,4^, Kunyu Xu^5^, Tianye Jia^1, 2^, Wei Zhou^6^, Colin Blakemore^7*^, Li-Hai Tan^8, 9*^, Miao Cao^1, 2*^, and Jianfeng Feng^1, 2^

^1^ Institute of Science and Technology for Brain-Inspired Intelligence, Fudan University, Shanghai 200433,

China

^2^ Key Laboratory of Computational Neuroscience and Brain-Inspired Intelligence (Fudan University), Ministry of Education, China

^3^ Department of Computer Science, University of Warwick, Coventry CV4 7AL, UK

^4^ Oxford Centre for Computational Neuroscience, Oxford, UK

^5^ Institute of Modern Languages and Linguistics, Fudan University, Shanghai 200433, China

^6^ Beijing Key Laboratory of Learning and Cognition, School of Psychology, Capital Normal University, Beijing 100037, China

^7^ Department of neuroscience, City University of Hong Kong, Hong Kong 999077, China

^8^Guangdong-Hongkong-Macau Institute of CNS Regeneration and Ministry of Education CNS Regeneration Collaborative Joint Laboratory, Jinan University, Guangzhou 510632, China

^9^ Center for Language and Brain, Shenzhen Institute of Neuroscience, Shenzhen 518057, China.

* Correspondence to: Miao Cao ([mcao@fudan.edu.cn](mailto:mcao@fudan.edu.cn)), Jianfeng Feng (jianfeng64@gmail.com) and Lihai Tan (tanlh@sions.cn)

**Supplementary Figures**


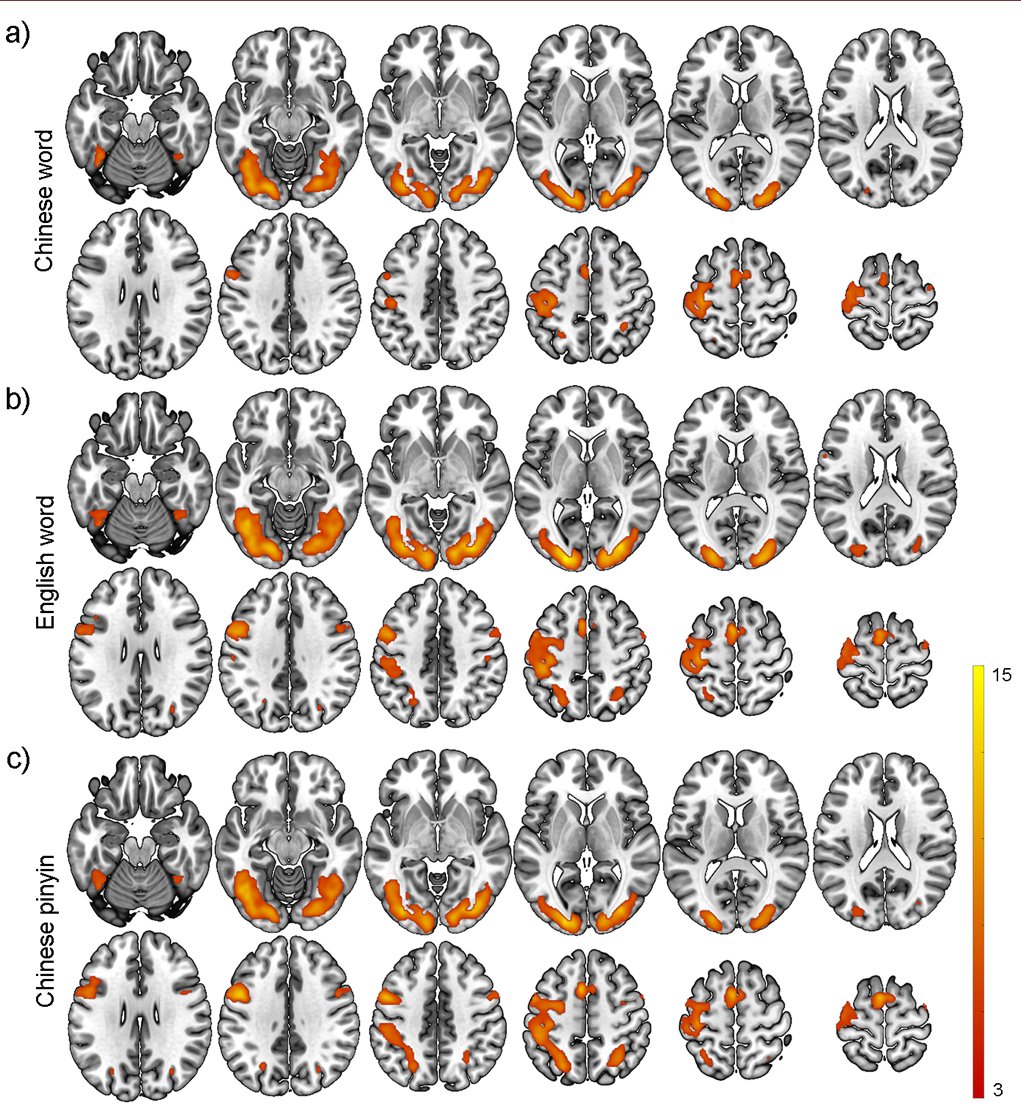


**Supplementary Figure 1. Validation of activation analysis for Chinese word, English word and Chinese pinyin reading tasks.** Second-level general linear models (GLMs) were performed separately to obtain activation maps for the recognition of **a).** Chinese words, **b).** English words and **c).** Chinese pinyin**.** Brighter colors indicate higher t values. (Voxel-wise p < 0.05, Bonferroni corrected p < 0.05 and cluster size > 10).


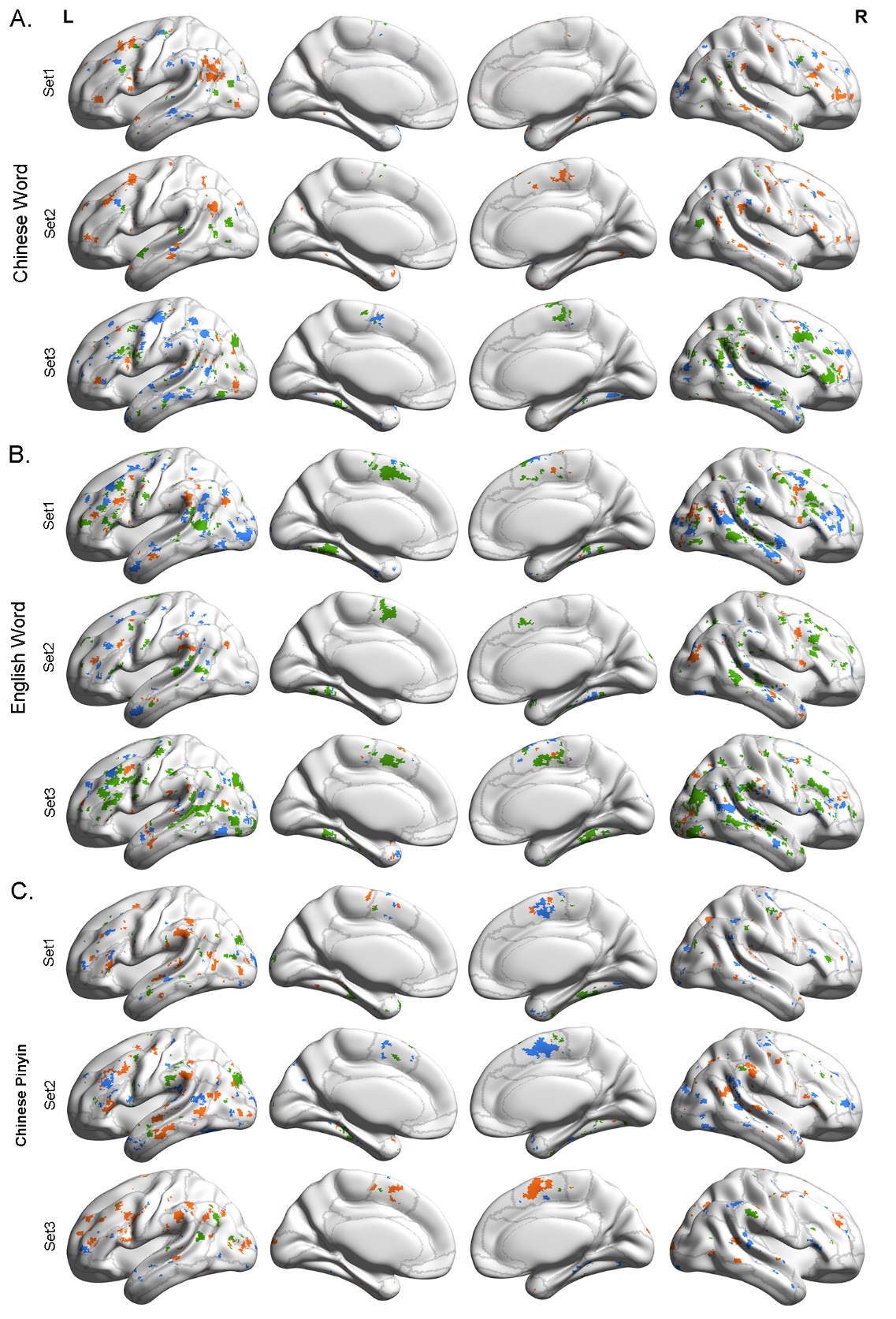


**Supplementary Figure 2. Validation of the brain activations for three linguistic components across languages.** Brain involvement maps of three linguistic components for **a).** Chinese words, **b).** English words and **c).** Chinese pinyin based on three data-sets. Data-set 1: 41 participants with right handedness; Data-set 2: randomly selected 21 participants with right handedness; Data-set 3: the other 20 participants with right handedness. Blue indicates brain activity for logo-grapheme. Green represents brain activity for phonology, and orange denotes brain activity for semantics. Grey lines indicate the regional boundaries in AAL.


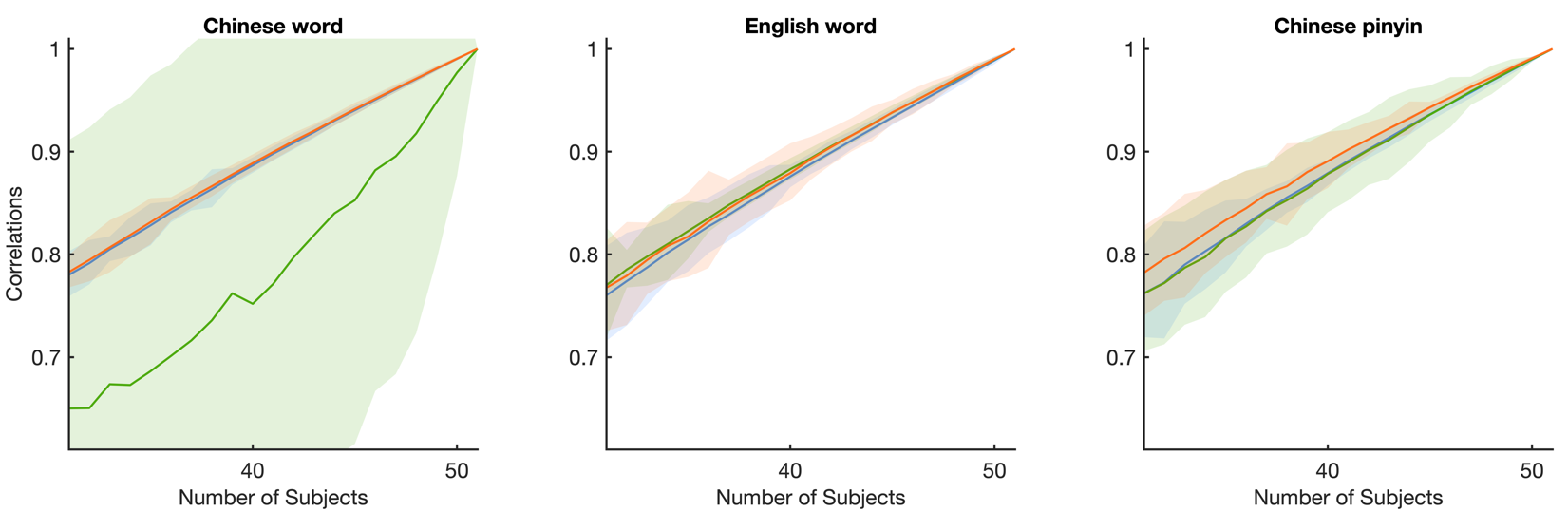


**Supplementary Figure 3. Validation of the spatial correlations in linguistic representation maps between randomly-selected subgroups and all participants.** The subgroup participants were randomly selected from all participants with the number ranging from 31 to 51 for 1000 times. Pearson correlation was calculated to scale the spatial similarity for **a).** Chinese words, **b).** English words and **c).** Chinese pinyin between the linguistic representation maps for each subgroup and all participants. Solid line indicated average correlation and colored area showed confidence intervals. The blue line is for the logo-grapheme component, the green line for the phonology component, and the red line for the semantic component.


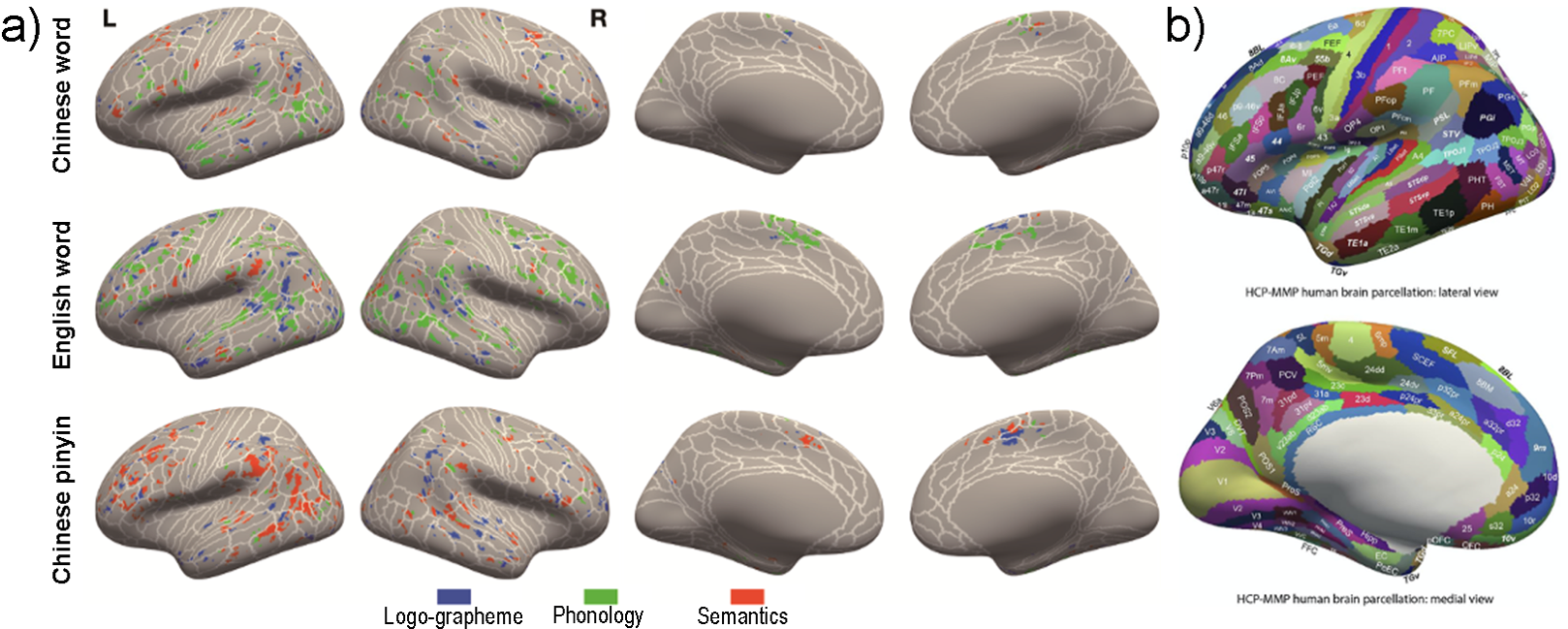


**Figure S4. Results illustrated on the HCPex template.** Panel **a)** shows brain involvement maps of three linguistic components for Chinese words, English words, and Chinese pinyin mapped on the Human Connectome Project Multimodal Parcellation (HCPex) template. White lines indicate the regional boundaries. The HCPex is illustrated in panel **b)** for better understanding of brain loads related to language components in the atlas. Permissions were obtained from the authors. For details, please see (Huang, Rolls, Feng &Lin, 2022).

**Supplementary Tables**

**Supplementary Table 1. Information of the overlaps in neural representations between languages.**

|  |  | Number of Voxels | Brain Regions |
| --- | --- | --- | --- |
| Logo-grapheme | Chinese word ∩ Chinese pinyin | 56 | Temporal_Sup_R |
|  |  | 20 | Frontal_Inf_Tri_R(15), Frontal_Inf_Tri_R(5) |
|  |  | 18 | Parietal_Inf_R(16), SupraMarginal_R (2) |
|  |  | 10 | Precentral_R (7), Frontal_Sup_R (3) |
|  | Chinese word ∩ English word | 11 | Fusiform_R (11) |
|  |  | 39 | Temporal_Sup_R (30), Temporal_Mid_R (9) |
|  |  | 12 | Frontal_Mid_L (12) |
|  |  | 10 | Frontal_Inf_Tri_R |
|  | English word ∩ Chinese pinyin | 22 | Temporal_Mid_L (20), Temporal_Pole_Mid_L (2) |
|  |  | 12 | Occipital_Inf_L (8), Occipital_Mid_L (4) |
|  |  | 23 | Temporal_Sup_R (16), Temporal_Mid_R (7) |
|  |  | 18 | Temporal_Sup_R (15), Heschl_R (3) |
|  |  | 13 | Temporal_Sup_R (11), Temporal_Mid_R (2) |
|  |  | 10 | Angular_R (5) |
| Phonology | Chinese word ∩ Chinese pinyin | - | - |
|  | Chinese word ∩ English word | 19 | Temporal_Mid_R (19) |
|  |  | 19 | Temporal_Mid_R (19) |
|  |  | 50 | Temporal_Mid_L (29), Temporal_Sup_L (21) |
|  |  | 10 | Occipital_Mid_R (10) |
|  |  | 25 | Temporal_Mid_L (25) |
|  |  | 70 | Temporal_Mid_L (37), Temporal_Sup_L (33) |
|  |  | 30 | Occipital_Mid_R (18), Temporal_Mid_R (12) |
|  |  | 12 | Occipital_Sup_L (12) |
|  |  | 11 | Frontal_Inf_Oper_R (8), Frontal_Inf_Tri_R (2), Frontal_Mid_R (1) |
|  |  | 15 | Frontal_Mid_R (10), Precentral_R (4) |
|  |  | 21 | Supp_Motor_Area_R (21) |
|  | English word ∩ Chinese pinyin | 15 | Fusiform_R |
|  |  | 39 | Occipital_Sup_L (26), Occipital_Mid_L (12) |
|  |  | 17 | SupraMarginal_R (17) |
|  |  | 33 | Precentral_R (33) |
|  |  | 20 | Parietal_Sup_R (9), Parietal_Inf_R (6), Postcentral_R (5) |
| Semantic | Chinese word ∩ Chinese pinyin | 15 | Frontal_Inf_Tri_L (11), Frontal_Mid_L (4) |
|  |  | 16 | Temporal_Mid_R (16) |
|  |  | 52 | Temporal_Mid_L (32), Angular_L (18) |
|  | Chinese word ∩ English word | 21 | Precentral_L (21) |
|  | English word ∩ Chinese pinyin | 11 | Frontal_Inf_Tri_L (11) |
|  |  | 12 | SupraMarginal_L (9), Postcentral_L (3) |
|  |  | 63 | SupraMarginal_L (60), Parietal_Inf_L (3) |

The brain regions are from the AAL atlas (Tzourio-Mazoyer et al., 2002).

**Supplementary Table 2. Correlations of neural representation maps between right-handedness only and all participants.**

|  | Chinese words | | | English words | | | Chinese pinyin | | |
| --- | --- | --- | --- | --- | --- | --- | --- | --- | --- |
|  | Logo-grapheme | Phonology | Semantic | Logo-grapheme | Phonology | Semantic | Logo-grapheme | Phonology | Semantic |
| *r* | 0.888 | 0.895 | 0.896 | 0.831 | 0.829 | 0.842 | 0.831 | 0.841 | 0.861 |
| *p* | 0 | 0 | 0 | 0 | 0 | 0 | 0 | 0 | 0 |

**Supplementary Table 3. Cognitive loads defined as the summed number of correlation values elicited by each linguistic component with RSA analysis in each ROI across languages.**

|  | Chinese word | | | English word | | | | Chinese pinyin | | |
| --- | --- | --- | --- | --- | --- | --- | --- | --- | --- | --- |
|  | Logo-grapheme | Phonology | Semantic | Logo-grapheme | | Phonology | Semantic | Logo-grapheme | Phonology | Semantic |
| Frontal_Mid_L | 1.34 | 0.61 | 5.37 | 9.7 | 7.91 | | 1.02 | 3.34 | 0.71 | 18.73 |
| Frontal_Mid_R | 3.42 | 1.94 | 10.66 | 1.5 | 16.71 | | 0.87 | 5.15 | 0.48 | 3.97 |
| Frontal_Inf_Oper_L | 0.18 | 1.27 | 1.63 | 0.14 | 1.51 | | 0.55 | 0.23 | 0.14 | 2.75 |
| Frontal_Inf_Oper_R | 1.56 | 3.42 | 0.56 | 0.5 | 5.5 | | 1.05 | 0.95 | 0.04 | 0.53 |
| Frontal_Inf_Tri_L | 0.41 | 3.15 | 1.97 | 2.8 | 8.22 | | 2.07 | 3.77 | 0.06 | 12.86 |
| Frontal_Inf_Tri_R | 3.73 | 1.25 | 0.5 | 1.05 | 8.24 | | 0.05 | 2.41 | 0.93 | 4.62 |
| Precentral_L | 3.22 | 2.92 | 1.39 | 3.44 | 13.84 | | 2.58 | 0.64 | 0.97 | 6.95 |
| Precentral_R | 1.88 | 1.46 | 1.51 | 0.93 | 12.6 | | 0.94 | 1.22 | 1.95 | 2.06 |
| Supp_Motor_L | 0.86 | 0.66 | 0.18 | 1.71 | 18.21 | | 0.33 | 2.15 | 0.86 | 4.26 |
| Supp_Motor_R | 0.34 | 2.24 | 2.57 | 3.86 | 8.81 | | 0.41 | 6.57 | 0.73 | 4.78 |
| Parietal_Inf_L | 1.03 | 0.12 | 1.06 | 2.32 | 8.94 | | 0.97 | 3.61 | 0.31 | 6.54 |
| Parietal_Inf_R | 1.79 | 0.56 | 0.24 | 0.26 | 1.77 | | 0.18 | 3.25 | 1.06 | 0.4 |
| SupraMarginal_L | 1.16 | 0.75 | 0.22 | 0.63 | 1.01 | | 3.57 | 0.95 | 0.28 | 7.43 |
| SupraMarginal_R | 0.18 | 1.61 | 1.15 | 0.67 | 6.4 | | 0.39 | 1.61 | 0.67 | 3.34 |
| Angular_L | 1.49 | 0.02 | 1.6 | 2.85 | 1.14 | | 0.49 | 0.39 | 0.37 | 3.97 |
| Angular_R | 0.52 | 1.05 | 0.48 | 1.41 | 3.46 | | 1.31 | 1.68 | 0.18 | 1.57 |
| Temporal_Sup_L | 1.5 | 3.82 | 2.57 | 2.16 | 7.86 | | 0.88 | 0.97 | 0.58 | 2.84 |
| Temporal_Sup_R | 4.21 | 1.08 | 0.46 | 4.27 | 7.92 | | 0.89 | 8.12 | 0.16 | 6.02 |
| Temporal_Pole_L | 2.4 | 1.31 | 0.52 | 3.59 | 1.88 | | 0.63 | 1.6 | 0.58 | 0.89 |
| Temporal_Pole_R | 1.85 | 1.44 | 0.64 | 1.18 | 2.45 | | 1 | 1.41 | 0.42 | 1.86 |
| Temporal_Mid_L | 3.63 | 9.95 | 3.89 | 9.21 | 16.89 | | 2.83 | 5.14 | 1.01 | 22.53 |
| Temporal_Mid_R | 2.47 | 5 | 4.05 | 6.08 | 14.52 | | 0.55 | 6.24 | 0.27 | 6.88 |
| Fusiform_L | 1.18 | 0.44 | 0.35 | 1.41 | 8.15 | | 0.96 | 1.83 | 1.47 | 1.35 |
| Fusiform_R | 1.74 | 1.26 | 1.75 | 2.49 | 5.89 | | 0.73 | 2.14 | 2.63 | 1.79 |
| Occipital_Sup_L | 0.2 | 0.43 | 0.86 | 0.13 | 4.33 | | 0.12 | 2.23 | 1.78 | 2.4 |
| Occipital_ Sup_R | 1.59 | 2.89 | 0.69 | 4.8 | 7.58 | | 1.76 | 2.23 | 1.44 | 9.34 |
| Occipital_ Mid_L | 0.35 | 1.23 | 0.49 | 2.36 | 3.27 | | 0.56 | 1.83 | 0 | 0.4 |
| Occipital_Mid_R | 1.2 | 0.31 | 0 | 0.86 | 4.8 | | 0.25 | 0.63 | 0.58 | 6.49 |
| Occipital_Inf_L | 1.53 | 1.39 | 0.36 | 0.75 | 14.75 | | 6.32 | 1.66 | 0.08 | 3.02 |
| Occipital_Inf_R | 0.11 | 0 | 0.03 | 0.91 | 5.04 | | 1.1 | 1 | 0 | 0.54 |

The brain regions are from the AAL atlas (Tzourio-Mazoyer et al., 2002).

**Supplementary Table 4. Cognitive loads defined as the summed number of significant voxels elicited by each linguistic component with RSA analysis in each ROI across languages.**

|  | Chinese word | | | English word | | | Chinese pinyin | | |
| --- | --- | --- | --- | --- | --- | --- | --- | --- | --- |
|  | Logo-grapheme | Phonology | Semantic | Logo-grapheme | Phonology | Semantic | Logo-grapheme | Phonology | Semantic |
| Frontal_Mid_L | 41 | 19 | 150 | 280 | 213 | 29 | 88 | 18 | 460 |
| Frontal_Mid_R | 102 | 57 | 282 | 41 | 430 | 21 | 143 | 14 | 104 |
| Frontal_Inf_Oper_L | 6 | 37 | 45 | 4 | 39 | 16 | 6 | 4 | 65 |
| Frontal_Inf_Oper_R | 47 | 100 | 16 | 14 | 139 | 29 | 25 | 1 | 13 |
| Frontal_Inf_Tri_L | 11 | 94 | 55 | 78 | 210 | 55 | 92 | 2 | 303 |
| Frontal_Inf_Tri_R | 115 | 39 | 14 | 29 | 203 | 1 | 60 | 21 | 110 |
| Precentral_L | 90 | 80 | 39 | 90 | 328 | 69 | 15 | 23 | 154 |
| Precentral_R | 53 | 42 | 43 | 23 | 315 | 23 | 32 | 48 | 52 |
| Supp_Motor_L | 24 | 20 | 5 | 46 | 436 | 10 | 56 | 21 | 98 |
| Supp_Motor_R | 10 | 66 | 69 | 105 | 225 | 13 | 161 | 20 | 119 |
| Parietal_Inf_L | 30 | 4 | 30 | 64 | 224 | 28 | 86 | 8 | 157 |
| Parietal_Inf_R | 56 | 19 | 7 | 7 | 44 | 4 | 77 | 27 | 9 |
| SupraMarginal_L | 33 | 24 | 6 | 18 | 28 | 96 | 22 | 7 | 170 |
| SupraMarginal_R | 6 | 50 | 33 | 19 | 168 | 11 | 41 | 20 | 79 |
| Angular_L | 45 | 1 | 46 | 77 | 30 | 14 | 10 | 12 | 111 |
| Angular_R | 16 | 34 | 13 | 42 | 86 | 39 | 45 | 5 | 44 |
| Temporal_Sup_L | 47 | 115 | 66 | 62 | 209 | 24 | 25 | 16 | 68 |
| Temporal_Sup_R | 129 | 33 | 13 | 123 | 216 | 25 | 203 | 5 | 149 |
| Temporal_Pole_L | 74 | 40 | 15 | 104 | 52 | 17 | 43 | 14 | 22 |
| Temporal_Pole_R | 57 | 42 | 17 | 34 | 68 | 27 | 37 | 12 | 47 |
| Temporal_Mid_L | 102 | 295 | 112 | 250 | 438 | 75 | 129 | 28 | 544 |
| Temporal_Mid_R | 77 | 145 | 117 | 166 | 377 | 15 | 162 | 8 | 174 |
| Fusiform_L | 35 | 14 | 9 | 39 | 203 | 27 | 42 | 38 | 34 |
| Fusiform_R | 54 | 37 | 49 | 65 | 145 | 18 | 52 | 69 | 44 |
| Occipital_Sup_L | 6 | 12 | 23 | 5 | 105 | 3 | 50 | 41 | 53 |
| Occipital_ Sup_R | 44 | 84 | 18 | 111 | 175 | 43 | 47 | 34 | 197 |
| Occipital_ Mid_L | 9 | 34 | 11 | 60 | 73 | 13 | 40 | 0 | 9 |
| Occipital_Mid_R | 31 | 9 | 0 | 22 | 110 | 5 | 14 | 14 | 140 |
| Occipital_Inf_L | 38 | 42 | 10 | 20 | 342 | 151 | 39 | 2 | 66 |
| Occipital_Inf_R | 3 | 0 | 1 | 20 | 102 | 25 | 20 | 0 | 10 |

The brain regions are from the AAL atlas (Tzourio-Mazoyer et al., 2002).

**Supplementary Table 5. Correlation between linguistic RDMs across languages.**

|  | Chinese word | | | English word | | | Chinese pinyin | | |
| --- | --- | --- | --- | --- | --- | --- | --- | --- | --- |
|  | Logo-grapheme | Phonology | Semantic | Logo-grapheme | Phonology | Semantic | Logo-grapheme | Phonology | Semantic |
| Logo-grapheme | - |  |  | - |  |  | - |  |  |
| Phonology | 0.048 | - |  | 0.371*** | - |  | 0.598*** | - |  |
| Semantic | -0.007 | 0.017 | - | 6.283e-04 | 0.047 | - | 0.109** | 0.109** | - |

* indicates p<0.01; *** indicates p < 0.001, Bonferroni-corrected

**References:**

Huang, C.-C., Rolls, E. T., Feng, J., & Lin, C.-P. (2022). An extended Human Connectome Project multimodal parcellation atlas of the human cortex and subcortical areas. Brain Structure and Function, 227(3), 763-778.

Tzourio-Mazoyer, N., Landeau, B., Papathanassiou, D., Crivello, F., Etard, O., Delcroix, N., . . . Joliot, M. (2002). Automated anatomical labeling of activations in SPM using a macroscopic anatomical parcellation of the MNI MRI single-subject brain. *Neuroimage, 15*(1), 273-289.
