# Supplementary material for: Intersecting distributed networks support convergent linguistic functioning across different languages in bilinguals
Source: Commun Biol. 2023 Jan 25;6:99. doi: 10.1038/s42003-023-04446-5 (PMC9876897; doi:10.1038/s42003-023-04446-5)
Supplement: Supplementary file 3 — Description of Additional Supplementary Files [file 42003_2023_4446_MOESM3_ESM.pdf]

Description of Additional Supplementary Files

File name: Supplementary Data 1

Description: Cognitive loads defined as the summed number of significant voxels for Chinese word in HCPex template.

File name: Supplementary Data 2

Description: Cognitive loads defined as the summed number of significant voxels for English word in HCPex template.

File name: Supplementary Data 3

Description: Cognitive loads defined as the summed number of significant voxels for Chinese pinyin in HCPex template.
